# Exploring Drug Use and Healthcare Utilization Among Adult Suicide Attempters: A Decision Tree Approach Using National Survey Data

**DOI:** 10.1155/jonm/4823915

**Published:** 2025-12-23

**Authors:** Bohyun Choi, Dajung Ryu, Mihyeon Seong, Sohyune Sok

**Affiliations:** ^1^ Health Insurance Review & Assessment Service, 3th, 13, Doum 4-ro, Sejong-si, 30113, Republic of Korea; ^2^ Department of Nursing, Kyungmin University, Uijeongbu-si, Gyeonggi-do, Republic of Korea, kyungmin.ac.kr; ^3^ Department of Nursing, University of Kaya, Gimhae-si, Gyeongnam, 50830, Republic of Korea; ^4^ College of Nursing Science, Kyung Hee University, Seoul, Republic of Korea, khu.ac.kr

**Keywords:** decision tree, medical experiences, medication, suicide attempters

## Abstract

**Background:**

Korea has one of the highest suicide rates among OECD countries, ranking first for several consecutive years, with a rate of approximately 24 deaths per 100,000 population. Understanding the association between prescription drugs and suicide attempts is crucial for developing effective suicide prevention and management strategies. This study analyzed Health Insurance Review and Assessment Service data to identify medical events preceding suicide attempts and to construct a classification model of prescribed medications using decision tree analysis.

**Methods:**

This secondary data study analyzed 1264 adult suicide attempters aged 20 to 59 years who were recruited from a pilot project for postmanagement in emergency departments conducted from March 2021 to March 2023, which may limit the generalizability of the findings. Claims data from the Health Insurance Review and Assessment Service were extracted using the specific item codes, and the drug details were obtained from the Ministry of Food and Drug Safety’s pharmaceutical classification table 100–890.

**Results:**

Of the total subjects, 65% were female and 35% male, with the highest proportion aged 20–29 years. Medications prescribed prior to suicide attempts were analyzed according to age‐specific prescription patterns, with a focus on the types and classes of drugs prescribed. The classification model revealed age‐specific patterns, with psychotropic medications emerging as major predictors across all age groups. These models included psychotropic medications, digestive ulcer drugs, antipyretic and analgesic agents, anticonvulsants, and respiratory system drugs, with respiratory medications emerging as the most crucial variable. In the 50s, psychotropic medications—particularly hypnotics and sedatives—appeared in all four pathways. Among these, zolpidem was most commonly prescribed for insomnia, underscoring the strong link between sleep disturbance and suicide risk. Of particular concern, nearly nine out of 10 suicide attempters were prescribed at least one medication carrying suicide‐related warnings or contraindications, as noted by the Ministry of Food and Drug Safety and the U.S. Food and Drug Administration.

**Conclusion:**

The results highlight differences in healthcare utilization and prescribed medication patterns by age group among suicide attempters.

**Implications for nursing management:**

These results can be used as foundational data for considering suicide risk during nursing assessment and diagnosis of general and psychiatric patients and developing specialized nursing strategies and management.

**Summary**

• Implications for the profession and/or patient care:
◦ There is potential for the development of a computer‐assisted suicide risk screening tool based on medication profiles.
◦ These findings suggest that prescription data can serve as a practical and scalable resource for identifying high‐risk populations.
◦ By incorporating these classification models into routine nursing workflows (especially in emergency department and psychiatric settings), nurses can improve suicide risk screening and provide timely intervention management.

## 1. Introduction

Suicide is a major global public health issue that profoundly affects individuals, families, communities, and nations across all regions, not only in high‐income countries. According to the World Health Organization (WHO), approximately 727,000 people die by suicide each year, making it one of the leading preventable causes of death worldwide [1]. Within the Organization for Economic Co‐operation and Development (OECD), South Korea reports the highest suicide rate—24.3 deaths per 100,000 population as of 2021 [2]. Given Korea’s universal health insurance system, which enables comprehensive monitoring of healthcare utilization and prescription data, the country provides a unique context for investigating the association between prescribed medications and suicide risk. This ongoing public health crisis underscores the need for comprehensive and evidence‐based prevention strategies.

Suicide results from complex interactions among biological, psychological, and social factors. Among these, medication‐related factors—such as the use of psychotropic drugs and polypharmacy—have recently gained increasing attention as potential risk indicators [3, 4]. Previous suicide attempts are recognized as the strongest predictor of subsequent suicide [1]. In addition, multiple risk factors have been consistently identified in the literature. **Substance dependence**, including alcohol and drug misuse, has been associated with a two‐ to four‐fold higher risk of suicidal ideation and attempts [3]. **Economic stressors**, such as unemployment, debt, and financial hardship, also contribute substantially to suicide risk [4]. Furthermore, **chronic pain and physical illness** have emerged as significant predictors, with individuals experiencing persistent pain conditions being two to three times more likely to attempt suicide [5]. **Gender differences** are also notable: Women are more likely to attempt suicide, whereas men have higher rates of suicide completion, highlighting the importance of gender‐specific interventions [6]. The **characteristics of suicide attempters** extend beyond mental health diagnoses, encompassing psychiatric comorbidities, socioeconomic disadvantages, chronic physical conditions, and patterns of healthcare utilization prior to the attempt [7]. Such findings indicate that suicide attempts cannot be explained by psychiatric illness alone but must be understood as the outcome of intertwined psychological, medical, and social determinants. More recently, attention has been directed toward physical illnesses and prescribed medications as indirect indicators of suicide risk. In particular, psychotropic medications such as antidepressants, anxiolytics, and hypnotics have been the subject of regulatory warnings and empirical studies linking their use to altered suicide risk, especially during the early phases of treatment [8–10]. These findings highlight the importance of monitoring prescription patterns when assessing suicide vulnerability. In South Korea, where the national health insurance system covers the vast majority of medications, prescription data provide particularly valuable insights into both physical and mental health status. Chronic conditions requiring long‐term pharmacological treatment can increase suicide risk, not only because of the disease burden itself but also due to associated symptoms such as depression, hopelessness, or loss of social roles [5]. Thus, suicide prevention requires interpreting demographic, clinical, and psychosocial risk factors in the broader context of suicide behavior progression, while also considering systemic and nursing perspectives.

Although many previous studies on suicide have revealed associations between mental illness, substance use, and suicide attempts [6], there is still a lack of research examining how **prescribed medications** contribute to suicidal behaviors. Most prior studies have focused on specific drug classes, such as antipsychotics, and their relationship with suicide risk [7]. Moreover, few have adopted advanced analytic methods to capture complex patterns of risk across multiple variables.

To address this gap, the present study aims to develop a suicide attempt prediction model using decision tree analysis, a data mining method that classifies and predicts outcomes through rule‐based hierarchical structures. Compared with conventional regression models, decision trees provide superior clinical interpretability by visually illustrating the interactions and relative importance of predictors. This transparency enhances the understanding of complex risk patterns and allows healthcare professionals to apply the findings directly to clinical decision‐making. Furthermore, this approach enables the identification of key predictors and high‐risk profiles by integrating demographic characteristics, healthcare utilization, and prescribed medications, while uncovering latent patterns within large‐scale healthcare data [11]. The data were obtained from claims for postmanagement services submitted to the Health Insurance Review and Assessment Service (HIRA), as part of a national pilot project for suicide attempters treated in emergency departments between March 2021 and March 2023. Accordingly, the specific objectives of this study are to (1) identify the demographic and clinical characteristics of the subjects, including patterns of healthcare service utilization; (2) assess perceived needs and unmet demands for healthcare services among suicide attempters; (3) compare prescribed medication profiles according to suicide attempt methods, frequency, or severity; and (4) develop and validate age‐specific classification models for suicide attempts based on patterns of prescribed medication use.

## 2. Methods

### 2.1. Design

This study employed a retrospective observational design based on secondary data analysis. The dataset consisted of healthcare claims submitted to the HIRA for postattempt management of individuals who had attempted suicide. The data spanned from March 2021 to March 2023 and included demographic information, healthcare service utilization, and prescribed medication records. While this approach enabled the analysis of large‐scale, real‐world data, it inherently limited the inclusion of psychosocial or behavioral variables that are not captured in administrative datasets.

### 2.2. Study Subjects

#### 2.2.1. Project Context and Data Source

This study utilized data from a national pilot project aimed at enhancing postattempt care for individuals who attempted suicide. The dataset was extracted from claims submitted to the HIRA, and included the following pilot project billing codes:

ID010: Referral patient management fee

ID020: In‐depth assessment fee

ID030: Case management plan establishment fee

ID040: Emergency observation management fee for suicide attempters

These codes were used to identify eligible cases and define the scope of services provided, and served both as inclusion criteria and as variables for analysis of healthcare service utilization.

#### 2.2.2. Subjects

The study population consisted of 1264 individuals aged 20–59 years who attempted suicide and received care at medical institutions participating in the pilot project. Subjects were included if their claims data contained one or more of the aforementioned billing codes, indicating engagement in postattempt clinical management. Demographic variables (e.g., age and sex) and clinical data (e.g., diagnosis and prescribed medications) were extracted for further analysis. Cases with missing demographic information, incomplete claims, or duplicate records were excluded from the analysis to ensure data accuracy and consistency.

### 2.3. Data Collection

The data used in this study were secondary claims data obtained from the HIRA. These claims include patient demographic information (e.g., age and sex), clinical information (e.g., diagnosis codes and service utilization), and prescription records associated with suicide attempters who received care under the national pilot project. The data period covered by the dataset spans from March 2021 to March 2023, corresponding to the operational timeline of the pilot project. The analysis of this dataset was conducted between March 2023 and January 2024.

Prior to the analysis, a preliminary literature review was conducted to inform the categorization of relevant variables. Based on this review, clinical characteristics and service records were grouped by suicide‐related risk factors, such as psychiatric history, emergency care utilization, and type of medical intervention. Additionally, all prescribed medications were classified according to the drug classification system provided by the Ministry of Food and Drug Safety (MFDS), which categorizes drugs based on their therapeutic class (e.g., antipsychotics, antidepressants, and sedatives). This classification enabled a structured analysis of medication use patterns among different risk groups.

### 2.4. Variables and Data Organization

As this study was based on secondary data analysis, variables were extracted from administrative claims submitted to the HIRA. These variables were organized into four primary categories: (1) general characteristics and healthcare utilization, (2) psychiatric diagnoses, (3) postattempt healthcare services provided, and (4) prescribed medications.

First, variables related to general characteristics and healthcare utilization included sex (male or female), age (categorized by decade), type of health insurance (e.g., National Health Insurance, Medical Aid), history of repeated suicide attempts within the past 3 years, and utilization of medical institutions. Repeated suicide attempts were identified based on the presence of multiple claims involving suicide‐related diagnosis or procedure codes. Medical institution utilization was defined as the number and type of healthcare facilities visited by each subject during the study period, such as emergency departments, psychiatric clinics, or primary care facilities.

Second, psychiatric diagnoses were identified using the Korean Standard Classification of Diseases, Seventh Revision (KCD‐7) codes, which are based on the International Classification of Diseases, 10th Revision (ICD‐10). These codes were used to capture underlying mental health conditions (e.g., depressive disorders, anxiety disorders, schizophrenia, and substance use disorders) that could influence suicide risk and healthcare utilization patterns.

Third, the postattempt healthcare service category captured medical interventions recorded after the index suicide attempt. These variables reflected the actual healthcare services provided and served as proxies to assess the level of clinical need and service delivery. Included in this category were psychotherapy, physical therapy, treatment for insomnia, treatment for sexually transmitted diseases (STDs), administration of general anesthesia or systemic intubation, treatment for brain injury, and prescriptions of psychotropic medications lasting more than 90 days. These services were documented in the claims data and represented the clinical response following suicide attempts.

Finally, prescribed medications were classified according to the Pharmaceutical Classification Table 100–890 of the MFDS (Table A1). Drugs were further organized using the Anatomical Therapeutic Chemical (ATC) classification system, with major categories including antidepressants, antipsychotics, anxiolytics, anticonvulsants, hypnotics and sedatives, opioid analgesics, antipyretic and anti‐inflammatory agents, antihypertensive agents, antidiabetic agents, medications for peptic ulcers and gastroesophageal reflux disease, and respiratory system drugs.

### 2.5. Data Analysis

The collected data were analyzed using R Version 4.3.3 for Windows and RStudio Version 2023.12.1–402, with machine learning modeling conducted using the tidymodels framework. The objective of the analysis was to construct age‐specific classification models to identify patterns associated with suicide attempt characteristics, based on prescribed medication use and other clinical variables. Since the dataset consisted exclusively of individuals who had attempted suicide and participated in the pilot project, all analyses were conducted within this population. Therefore, the phrase “general population” was not applicable and has been revised accordingly. The data were stratified by outcome variable defined as the type of suicide attempt (e.g., overdose, hanging, and self‐injury) and split into training and test sets in a 3:1 ratio to preserve class proportions across outcome categories.

The outcome variable in the decision tree model was the classification of suicide attempt type, and the predictor variables included age, sex, type of medical institution visited, psychiatric history, and categorized prescribed medications (e.g., psychotropics, sedatives, and antiepileptics), among others. Prior to model training, categorical variables were one‐hot encoded, and no normalization was applied since decision tree algorithms are invariant to monotonic transformations of numerical inputs. To optimize the model, a grid search approach was used to tune the decision tree’s hyperparameters, with 10 levels assigned to each of three selected parameters, resulting in a total of 1000 combinations (10 × 10 × 10). Model training was performed using 10‐fold cross‐validation within the training set to prevent overfitting and ensure generalizability. The optimized hyperparameters were then used to build the final classification model, which was evaluated on the test dataset. Performance metrics included the area under the receiver operating characteristic curve (ROC‐AUC), accuracy, sensitivity, specificity, and F1‐score. Additionally, feature importance was extracted to identify the most influential predictors associated with the classification of suicide attempt types.

Although ensemble‐based algorithms such as random forest and XGBoost often demonstrate higher predictive accuracy, a single decision tree model was deliberately selected in this study to enhance clinical interpretability and transparency. The rule‐based, hierarchical structure of decision trees allows healthcare professionals to intuitively understand the relationships between predictors and outcomes, facilitating translation of model results into practical clinical decision‐making and risk communication.

Finally, to address Aim 4, separate classification models were constructed for each age group (e.g., 20s, 30s, 40s, and 50s) to examine age‐specific patterns in suicide attempt characteristics. This allowed for a comparative analysis across age strata, enhancing the interpretability and relevance of the findings for targeted intervention development.

### 2.6. Ethical Considerations

This study was exempted from review by the Institutional Review Board of the HIRA, as it involved the analysis of deidentified secondary data collected for administrative purposes (approval no. 2023‐009; approval date: March 3, 2023). All data used in the study were derived from the participants who had provided informed consent for data sharing and participation in the national pilot project at the time of service enrollment by signing a standardized agreement form. Furthermore, all datasets used in the analysis were fully anonymized prior to access by the researchers, in accordance with relevant data protection and ethical guidelines.

## 3. Results

### 3.1. General Characteristics and Healthcare Utilization of Subjects

As shown in Table 1, approximately two‐thirds of suicide attempters were women, and the largest age group was those in their twenties. A small but notable proportion had multiple suicide attempts within the past 3 years. In terms of healthcare utilization, nearly all participants visited clinics and hospitals, and about one‐fifth visited psychiatric hospitals (Table 1).

**Table 1:** General characteristics and healthcare utilization of subjects (*n* = 1264).

Variables		*n*	%
Male (age, year)		444	35.1
20–29		150	11.9
30–39		99	7.8
40–49		100	7.9
50–59		95	7.5
Female (age, year)		820	64.9
20–29		367	29.1
30–39		181	14.3
40–49		159	12.6
50–59		113	8.9
Insurance			
Health insurance		1,101	87.1
Medical assistance		163	12.9
Frequency of additional suicide attempts within the last 3 years (time)	Male		
	2	24	3.3
	3	5	1.1
	Female		
	2	47	5.7
	3	15	1.8
	4	2	0.2
Medical institution utilization^∗^	Tertiary hospitals	1256	98.2
	General hospitals	1178	92.1
	Psychiatric hospitals	281	21.9
	Hospitals	1128	88.2
	Clinics	1277	99.9

^∗^Multiple response.

### 3.2. Requirements and Demands of Healthcare Service of the Subjects

As presented in Table 2, the most frequent services received were psychotherapy, physical therapy, and insomnia treatment, followed by treatment for STDs, anesthesia or systemic intubation, and brain injury care. About one in five participants had long‐term prescriptions (> 90 days) for psychotropic medications (Table 2). Long‐term psychotropic prescription like this (20%) is the most concerning point.

**Table 2:** Requirements and demands of healthcare service of the subjects.

Variables	Categories	*n^∗^* (%)
Psychotherapy	Male	354 (27.7)
	Female	710 (55.6)
Physical therapy	Male	352 (27.5)
	Female	677 (53.0)
Insomnia treatment	Male	250 (19.6)
	Female	578 (45.2)
Sexually transmitted diseases treatment	Male	69 (5.4)
	Female	366 (28.6)
General anesthesia/systemic intubation	Male	158 (12.3)
	Female	251 (19.6)
Brain injuries treatment	Male	113 (8.8)
	Female	143 (11.1)
Prescriptions of psychotropic drugs for more than 90 days	Male	82 (6.4)
	Female	144 (11.3)

^∗^Multiple responses.

### 3.3. Comparison of Drug Prescriptions Based on Suicide Attempts

Table 3 compares prescribed medications between suicide attempters and controls. Overall, the suicide attempt group showed significantly higher exposure to psychotropic drugs (*d* = 0.85, 95% CI = 0.78–0.92), which represented the largest effect size observed in this analysis. This strong association also extended to related medications, including hypnotics/sedatives (*d* = 0.75, 95% CI = 0.68–0.81) and anticonvulsants (*d* = 0.52, 95% CI = 0.45–0.59). Conversely, the control group had higher use of medications for physical conditions such as respiratory system drugs, peptic ulcer drugs, and antihypertensive agents. No significant differences were observed for arteriosclerosis or diabetes medications (Table 3).

**Table 3:** Comparison of drug prescriptions based on suicide attempts.

Prescribed medications	Suicide attempts		t	*p*	Cohen’s *d* (95% CI)
	Yes (*n* = 1264)	No (*n* = 3096)			
	Mean ± SD^∗^				
Respiratory system drugs	3.8 ± 15.8	33.3 ± 73.7	−21.12	< 0.001	−0.47 (−0.54, −0.40)
Antihypertensive agents	152.3 ± 472.8	220.8 ± 890.9	−3.29	0.001	−0.09 (−0.15, −0.02)
Central nervous system drugs	152.7 ± 560.0	55.0 ± 355.6	5.75	< 0.001	0.23 (0.16, 0.30)
Diabetes drugs	99.6 ± 389.5	116.3 ± 690.4	−1.01	0.313	−0.03 (−0.09, 0.04)
Arteriosclerosis drugs	169.5 ± 478.7	198.7 ± 754.5	−1.53	0.126	−0.04 (−0.11, 0.02)
Drugs for peptic ulcers	136.4 ± 289.8	320.6 ± 559.9	−14.22	< 0.001	−0.37 (−0.44, −0.31)
Antipyretic and anti‐inflammatory drugs	244.7 ± 446.5	309.7 ± 469.4	−4.30	< 0.001	−0.14 (−0.21, −0.08)
Hypnotics/sedatives	292.1 ± 514.2	34.5 ± 244.4	17.04	< 0.001	0.75 (0.68, 0.81)
Anticonvulsants	311.8 ± 575.2	58.0 ± 447.1	14.05	< 0.001	0.52 (0.45, 0.59)
Psychotropic drugs	1172.4 ± 1501.1	215.2 ± 925.9	21.09	< 0.001	0.85 (0.78, 0.92)

^∗^Dosage date. Medications were classified into mutually exclusive categories according to the MFDS and ATC systems. “Psychotropic drugs” include antidepressants, antipsychotics, and anxiolytics. “Hypnotics/sedatives” and “anticonvulsants” were analyzed as separate categories to avoid overlap with central nervous system (CNS) drugs, which comprise nonpsychotropic agents such as analgesics and muscle relaxants.

### 3.4. Age‐Specific Suicide Attempt Classification Models Based on Prescribed Medications

**Figure 1:**
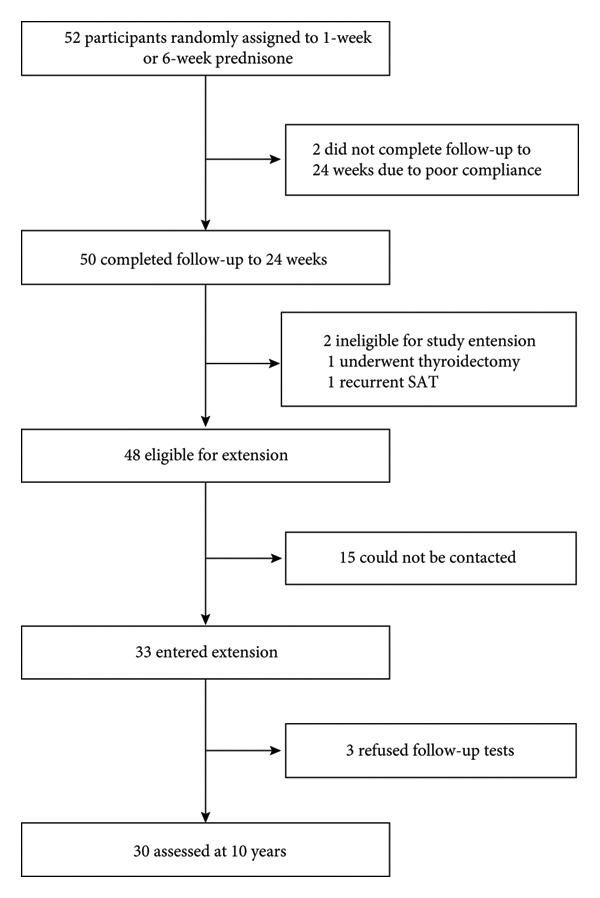
Suicide attempt classification model for age 20s (frequency).

**Figure 2:**
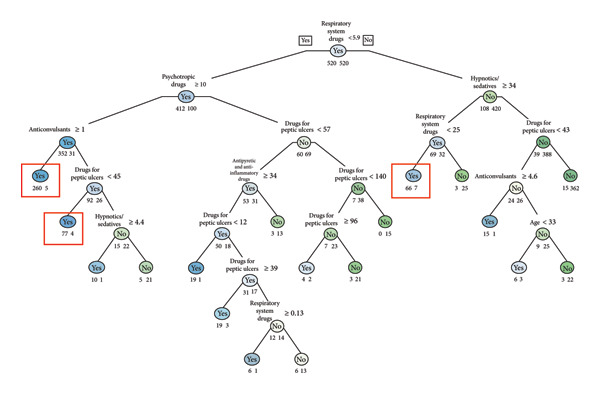
Suicide attempt classification model for age 30s (frequency).

**Figure 3:**
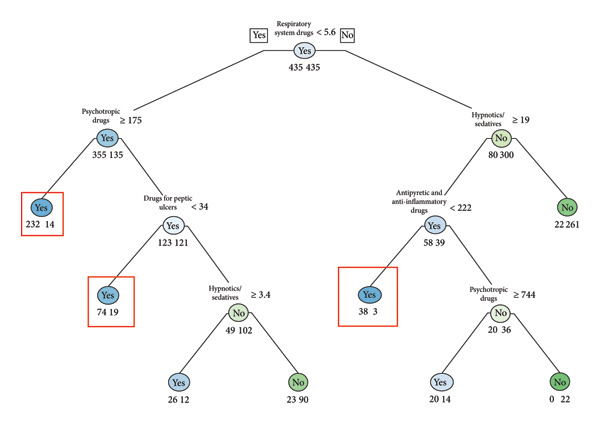
Suicide attempt classification model for age 40s (frequency).

**Figure 4:**
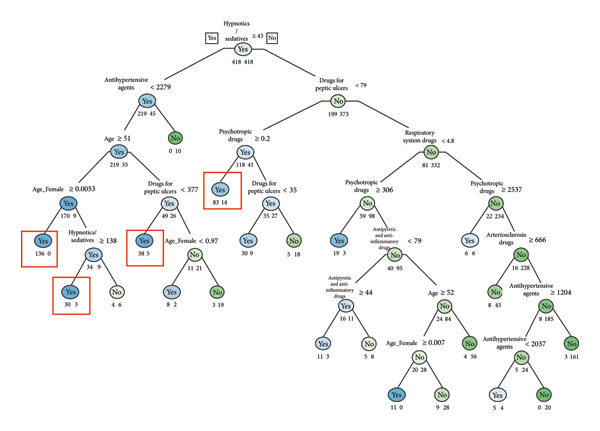
Suicide attempt classification model for age 50s (frequency).

Decision tree analyses (Figures 1, 2, 3, and 4) identified distinct age‐specific pathways for suicide attempts. In the **20s**, psychotropic medications were the strongest predictors, particularly prolonged prescriptions, with younger age further increasing risk. In the **30s and 40s**, combinations of psychotropic medications with drugs for chronic conditions (e.g., peptic ulcer drugs, antipyretic/anti‐inflammatory drugs, and respiratory drugs) were prominent. In the **50s**, hypnotics/sedatives appeared most consistently as predictors, with zolpidem frequently associated with suicide attempts. Gender‐specific patterns were also noted, with higher sedative use among women. The classification models achieved good predictive performance (AUC values 0.91–0.94 across age groups), highlighting the potential utility of prescription data for suicide risk screening (Figures 1, 2, 3, and 4). The high performance was further validated by the consistently strong F1‐scores—a key metric representing the balance between prediction precision and recall—ranging from 0.77 to 0.82 across all age groups. This demonstrates that the models maintain a high and balanced level of both sensitivity and specificity (Table A2).

## 4. Discussion

One of the most notable demographic features of this study was the disproportionately high proportion of suicide attempters in their twenties, accounting for 41% of the total participants. This pattern may reflect the implementation characteristics of the pilot project, which was concentrated in large urban hospitals serving younger populations. However, it may also indicate an actual increase in suicide attempts among younger adults, consistent with recent national trends reporting elevated suicide risk in this age group. Therefore, this finding should be interpreted with caution, considering both potential recruitment bias from urban pilot sites and the genuine vulnerability of young adults to suicide risk. It also contrasts with earlier findings by Chen et al. [7], who reported different age distributions, suggesting that regional disparities in healthcare accessibility and intervention availability may influence observed trends.

Another important finding was that 12% of suicide attempters were medical aid recipients, far exceeding the national average of 2.9%. This aligns with the evidence from Lee et al. [12] and international literature demonstrating that economic adversity and low socioeconomic status are strongly associated with depression, suicidal ideation, and attempts [13, 14]. Financial hardship thus remains a critical dimension of suicide prevention.

Of particular concern, nearly nine out of 10 suicide attempters in our cohort were prescribed at least one medication carrying suicide‐related warnings or contraindications, as indicated by regulatory agencies such as the MFDS and the U.S. Food and Drug Administration (FDA) [15, 16]. This finding underscores a critical clinical and pharmacological concern, suggesting that careful medication monitoring and risk assessment are essential components of suicide prevention strategies. These included psychotropic medications, sedative‐hypnotics, and anticonvulsants, all of which have been implicated in regulatory safety communications due to risks of psychiatric side effects, including increased suicidal ideation. Among psychotropic agents, multiple antidepressants carried explicit warnings, and international meta‐analyses confirm that the association between antidepressants and suicidality is age‐dependent particularly elevating risk in those under 25 during early treatment [9, 17, 18]. This underscores the necessity of age‐specific monitoring and individualized risk‐benefit assessments when initiating pharmacologic therapy for mental health conditions.

Our age‐stratified classification models provide further insights into these dynamics. In the twenties, prolonged prescriptions of psychotropic drugs emerged as strong predictors, even in the absence of chronic physical illnesses. This finding suggests the need for proactive monitoring systems such as electronic medical record (EMR) alerts to flag extended use of hypnotics or other psychotropic medications, enabling timely clinical review and screening of suicide risk. This suggests that psychiatric vulnerabilities manifest earlier and may require timely preventive interventions before physical comorbidities accumulate. In the thirties and forties, broader patterns emerged, with prescriptions for peptic ulcer treatments, antipyretic/anti‐inflammatory drugs, and respiratory medications, reflecting the well‐documented association between chronic physical conditions particularly chronic pain and elevated suicide risk [5, 13, 19]. Systematic reviews consistently report a two‐ to three‐fold increase in suicidal behavior among individuals with long‐standing pain conditions. In the fifties, sedative‐hypnotics, especially zolpidem and doxepin, appeared prominently. This is consistent with epidemiologic studies showing that prolonged use of sedative‐hypnotics, particularly among middle‐aged women, is associated with increased risk of depression and suicidal behavior [20, 21]. These findings highlight the importance of integrating both pharmacological and gender‐sensitive approaches into suicide prevention.

Placing our findings in the broader international literature, they align with global evidence that suicidality is multifactorial, influenced by psychiatric comorbidities, socioeconomic disadvantage, and physical morbidity [13, 14, 22]. Franklin et al. [13] and Zalsman et al. [14] emphasize that effective suicide prevention must integrate demographic, social, and medical risk factors, while Molero et al. [18] demonstrated population‐level associations between gabapentinoids and self‐harm. Our results extend these findings by demonstrating how routinely collected prescription data can serve as scalable markers for identifying high‐risk groups in specific age strata, offering a bridge between pharmacoepidemiology and nursing practice.

The implications for nursing and clinical practice are significant, highlighting the importance of integrating medication surveillance and suicide risk assessment into routine care. Moreover, these findings have interdisciplinary relevance for psychiatrists, primary care providers, and emergency physicians, supporting collaborative approaches to early identification and management of individuals at suicide risk. By incorporating prescription data into routine risk screening, nurses particularly in emergency and psychiatric settings can better identify vulnerable groups and provide timely interventions, e.g., prolonged hypnosedative use in midlife or extended psychotropic exposure in young adults could serve as triggers for targeted risk assessment. The integration of computer‐assisted suicide risk screening tools based on medication profiles could further support timely, evidence‐based decision‐making in clinical settings. Nevertheless, several limitations must be acknowledged. The study relies on secondary claims data, which lack detailed contextual information such as psychiatric assessments, psychosocial stressors, or patient‐reported outcomes. Thus, while prescription data provide valuable proxies, they cannot capture the full complexity of suicidality. Prospective studies with richer clinical and psychosocial data are necessary to validate and refine the predictive models developed here. Despite these limitations, the study underscores the value of prescription data as a practical, scalable resource for suicide risk identification. By refining age‐specific classification models and validating them across diverse populations, future research can contribute to the development of standardized, nurse‐led suicide prevention protocols that combine pharmacologic monitoring with holistic care.

### 4.1. Limitations

This study utilized data from a pilot project aimed at postattempt management of suicide attempters in South Korea. While the project was implemented nationwide, the dataset analyzed in this study reflects only a subset of the population, specifically, individuals who received services in medical institutions that voluntarily participated in the pilot project. Therefore, the findings should not be generalized to the entire Korean population. In addition to this limitation, several methodological constraints must be acknowledged. First, the use of secondary administrative data introduces the potential for information bias, as claims data are collected for billing purposes rather than for research. Important variables such as psychosocial context, severity of the suicide attempt (e.g., intent or lethality), psychiatric comorbidities, and the exact motivation behind medication use were not captured. Second, the study design is observational and cross‐sectional, which limits the ability to draw causal inferences between prescribed medications and suicide attempts. Although prescription data were extracted from the 12 months preceding the index suicide attempt, the temporal relationship between medication use and suicidal behavior remains uncertain. Third, although decision tree modeling was used to identify age‐specific risk profiles, the models were developed and tested using data from the same dataset, which raises concerns about model overfitting. Without external validation using an independent dataset, the generalizability and stability of the predictive models remain unconfirmed. Fourth, there is a risk of sampling bias due to the overrepresentation of certain demographic and geographic groups. In particular, individuals in their 20s accounted for a disproportionately high percentage of the sample (41%), and most data originated from urban medical institutions, which may limit the applicability of the findings to other age groups or rural populations. Future studies should address this imbalance through stratified sampling or inclusion of rural healthcare data.

Despite these limitations, the study provides an important exploratory analysis of prescription patterns and suicide risk using large‐scale national health data, and it offers a foundation for developing data‐driven nursing assessment tools. However, interpretation of the results should be made with caution, and further research is needed to confirm these findings in more diverse and representative populations.

## 5. Conclusion

Looking at the results of this study, in the recent three years from 2020 to 2023, the number of people and the number of times attempting suicide were higher for women than for men. The subjects used medical services in the following order: psychotherapy, physical therapy, insomnia treatment, STDs treatment, general anesthesia/intubation, brain injury treatment, and prescription of psychotropic drugs for more than 90 days. The suicide attempt group was found to have taken more psychotropic drugs, hypnotic sedatives, antiepileptics, and central nervous system drugs than the group that did not attempt suicide. Looking at the suicide attempt classification model by age group according to drug type, psychotropic drugs were used in the 20s, hypnotic sedatives and psychotropic drugs in the 30s, psychotropic drugs and hypnotic sedatives in the 40s, and hypnotic sedatives and psychotropic drugs in the 50s. Regardless of age, psychotropic drugs and hypnotic sedatives were most prevalent among individuals in their 20s–50s. These findings highlight that prescription data, particularly prolonged use of psychotropic and hypnotic medications, can serve as practical and scalable markers for suicide risk screening and early intervention within the Korean healthcare system.

## Ethics Statement

This study was exempted from the Institutional Review Board of the Health Insurance Review and Assessment Service (approval no. 2023‐009; approval date March 3, 2023).

## Conflicts of Interest

The authors declare no conflicts of interest.

## Funding

The authors received no specific funding for this work.

## Data Availability

The data that support the findings of this study are available from the corresponding author upon reasonable request.

## Appendix A

**Table A1:** Drug classification.

No.	Drug classification
110 drugs for central nervous system	111 general anesthetics
	112 hypnotics and sedatives
	113 anticonvulsants
	114 antipyretic, analgesic, and anti‐inflammatory agents
	115 stimulants
	116 antivertigo agents
	117 psychotropic agents
	119 other central nervous system drugs
120 drugs for peripheral nervous system	121 local anesthetics
	122 skeletal muscle relaxants
	123 autonomic nervous system agents
	124 antispasmodics
	125 diaphoretics and antiperspirants
	129 other peripheral nervous system drugs
∼	
210 drugs for circulatory system	211 cardiac stimulants (cardiotonic agents)
	212 antiarrhythmics
	213 diuretics
	214 antihypertensive agents
	215 vascular tonics
	216 vasoconstrictors
	217 vasodilators
	218 drugs for arteriosclerosis
	219 other circulatory system drugs
220 drugs for respiratory system	221 respiratory stimulants
	222 antitussives and expectorants
	223 inhalation agents
	229 other respiratory system drugs
230 drugs for digestive system	231 dental and oral agents
	232 drugs for peptic ulcers
	233 digestives and stomachics
	234 antacids
	235 emetics and antiemetics
	236 choleretics (bile secretion–promoting agents)
	237 antidiarrheal agents
	238 laxatives and purgatives
	239 other digestive system drugs
∼	
820 nonalkaloid narcotics	821 synthetic narcotics
	829 other nonalkaloid narcotics
890 other narcotics	

*Note:* Hypnotics and sedatives are a subgroup of psychotropic medications but were presented separately for analytical clarity.

**Table A2:** Model performance metrics by age group.

Metrics	Age group			
	20s	30s	40s	50s
AUC	0.91	0.94	0.93	0.94
Accuracy	0.86	0.87	0.88	0.86
Sensitivity	0.84	0.80	0.85	0.92
Specificity	0.86	0.90	0.90	0.84
Precision	0.72	0.77	0.79	0.68
Recall	0.84	0.80	0.85	0.92
F1‐score	0.77	0.78	0.82	0.78
